# Differences in Brain Activity After Learning With the Use of a Digital Pen vs. an Ink Pen—An Electroencephalography Study

**DOI:** 10.3389/fnhum.2019.00275

**Published:** 2019-08-09

**Authors:** Kiyoyuki Osugi, Aya S. Ihara, Kae Nakajima, Akiyuki Kake, Kizuku Ishimaru, Yusuke Yokota, Yasushi Naruse

**Affiliations:** ^1^Center for Information and Neural Networks, National Institute of Information and Communications Technology, Osaka University, Kobe, Japan; ^2^Graduate School of Frontier Bioscience, Osaka University, Suita, Japan; ^3^Wacom Co., Ltd., Kazo, Japan

**Keywords:** digital device, learning, electroencephalography, digital pen, handwriting

## Abstract

The purpose of this study is to clarify whether there is a learning effect on brain activity after writing with an ink pen vs. a digital pen. Previous studies have reported the superiority of handwriting to typing in terms of learning performance, but differences between the use of an ink pen vs. a digital pen remain unclear. In the present study, the participants learned to read difficult words by writing with an ink pen vs. a digital pen. After the learning period, electroencephalography (EEG) signals were measured, while the participants underwent a repetition priming paradigm with the use of the learned words. The repetition priming effect of the N400 event-related potential (ERP) was quantified as an index of the learning effect and the effects between pen types were compared. The groups were also subdivided according to whether a digital pen is frequently used (familiar vs. unfamiliar group). The number of writing repetitions for each word within 10 min during the learning activity and the post-learning test scores were not affected by the pen-type or familiarity with a digital pen. However, the repetition priming effect of the N400 was greater for words written with a digital pen in the learning session, as compared with an ink pen, in the familiar group, but not the unfamiliar group. These results suggest that for those familiar with its use, writing with a digital pen may improve learning relative to the use of an ink pen.

## Introduction

Digital devices are increasingly used in education, so understanding the differences in the effects on learning ability relative to the use of analog devices could lead to more effective educational practices. Experimental psychological studies have investigated the differences in learning effects between writing with a conventional pen and typing on a keyboard. In adult participants, recognition accuracy was higher after they learned unfamiliar characters by writing them down on paper than typing on a keyboard (Longcamp et al., 2008), and more words were recalled after writing on paper than typing (Mangen et al., 2015). Preschool children also learned letters and words more effectively by handwriting than typing (Longcamp et al., 2005; Kiefer et al., 2015). Additionally, handwriting seems to be more effective for conceptual comprehension than typing. In fact, comprehension assessment of listening to technology/entertainment/design talks was superior among college students who made notes in a notebook using a pen as compared to those who typed notes on a laptop computer (Mueller and Oppenheimer, 2014). The advantage of handwriting over typing has also been indicated in neuroscientific approaches using electroencephalography (EEG; van der Meer and van der Weel, 2017) and magnetic resonance imaging (Vinci-booher et al., 2016).

However, few studies have investigated whether learning effects differ between writing with a digital pen on a tablet vs. writing with a conventional pen on paper. Hatano et al. (2015) conducted an EEG experiment in which the participants took notes with a digital pen on a tablet or with a mechanical pencil on paper while listening to scientific lessons. There were no significant differences in the scores of comprehension and memory tests performed after taking notes on a tablet vs. paper. However, theta-band (4–7 Hz) EEG activity was higher when writing on a tablet than on paper. Because the theta-band EEG activity was reported to increase with the cognitive load (Gevins et al., 1997; Borghini et al., 2012; Anguera et al., 2014), the use of a digital pen and tablet require more cognitive effort to monitor written characters and more attention to movements for writing compared with writing on paper. In fact, recent studies reported that the movements of handwriting with a digital pen on a tablet are not the same as with a conventional pen on paper (Alamargot and Morin, 2015; Gerth et al., 2016a,b; Wollscheid et al., 2016; Guilbert et al., 2019). Alamargot and Morin (2015) demonstrated that the handwriting kinematics of school-age children when writing with a plastic-tipped pen on a tablet screen is different from when writing with a ballpoint pen on paper, and the effects vary with age, as second graders made longer pauses and nine graders increased pen pressure and speed. These results suggest that segment trajectory calculation is disturbed in younger children and control of muscular adjustment is disturbed in older children. From the points of view of movements and brain activities, handwriting with a digital pen on a tablet might disturb cognitive activities, such as learning. To the best of our knowledge, this is the first study to investigate whether brain activity after learning by handwriting with a digital pen on a tablet is different from that with a pen on paper. The difference of after-effect might vary according to the (un)familiarity with a digital device.

In the present study, the N400, an event-related potential (ERP) response, was used to measure the learning effect of handwriting with a digital pen vs. an ink pen. The N400 is a negative-going component peaking around 400 ms after exposure to words, pictures, and other meaningful stimuli (for a review, see Kutas and Federmeier, 2011), which is related to semantic processing so that the amplitude changes with the ease of accessing information from long-term memory and integrating semantic representations into a preceding context (for a review, see Kutas and Federmeier, 2000). Many studies have shown that the N400 changes with language learning (Ojima et al., 2005, 2011) and developmental progress (Friedrich and Friederici, 2004, 2010; Reid et al., 2009). Most importantly, the N400 effects have been observed in an earlier stage of learning than behavioral indices (McLaughlin et al., 2004). Regarding an adult’s ability to learn a second language (L2), McLaughlin et al. (2004) showed that the amplitude modulation of N400 discriminated between L2 words and pseudo-words after 14 h of classroom instruction, while the participants reached only chance levels when making overt L2 word-nonword judgments, suggesting that the N400 is a powerful tool to reveal the effects of learning, especially in the early stage.

**Figure 1:**
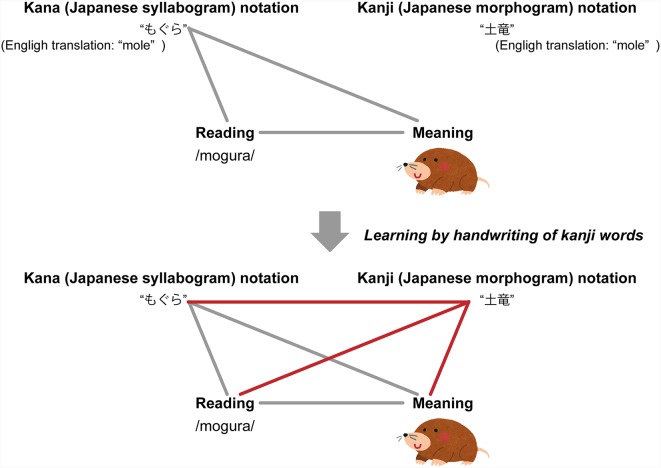
An example of a Japanese word written in kanji that is difficult to read. Mole in English corresponds to two notations in Japanese: “もぐら” written in kana (syllabogram) and “土竜” written in kanji (morphogram). However, the kana notation is generally used and most Japanese cannot read the kanji notation, so only the kana notation was associated with the reading and meaning of the word “mole,” but not the kanji notation. We assumed that in the learning activity, the participants wrote the kanji notations of such words, then the kanji notations have association with the reading and meaning, as well as the kana notation.

The Japanese language has two writing systems [i.e., kana (syllabograms) and kanji (morphograms)], and so, many words have two notations. As the learning content, well-known Japanese words are used that are generally written in kana (syllabograms), as most Japanese people cannot read. Although there are two notations, most are familiar with the kana notation, but not kanji notation (Figure 1). In the learning activity, the participants learned the readings of such words by writing with a digital pen on a tablet and with an ink pen on paper. Just after the learning activity, EEG experiments were conducted with a repetition priming paradigm to determine whether the repetition priming effects of the N400 were affected by learning tools (i.e., digital pen vs. ink pen) and/or familiarity with a digital pen and tablet. When two stimuli are presented consecutively and the subsequent stimulus (target) is identical/related to the preceding stimulus (prime), the N400 amplitude of the target decreases relative to the unrepeated/unrelated target (van Petten et al., 1991; Deacon et al., 2004; Matsumoto et al., 2005; Rugg, 1985; Holcomb, 1993). In the present study, the words written in the learning activity (prime) were followed by kana words (target) that were semantically and phonologically identical to the prime words (repetitive condition) or not (non-repetitive condition). We assumed that as learning progresses, a larger difference in the N400 amplitude between the repetitive and non-repetitive conditions (i.e., repetition priming effect of the N400) would occur. The differences in the repetition priming effect of the N400 with writing with a digital pen on a tablet vs. with an ink pen on paper were used for comparisons between participants familiar and unfamiliar with the digital pen system.

## Materials and Methods

### Participants

Twenty-eight healthy volunteers participated in the study. They were divided into two groups according to the results of a questionnaire distributed after the EEG experiment: 11 participants (10 men and one woman; age, 26–52 years) who used a digital pen/tablet system in their daily lives (familiar group) and 17 participants (10 men and seven women; age, 21–47 years) who did not (unfamiliar group). The study protocol was approved by the Bioinformatics Ethics Committee of the National Institute of Information and Communications Technology, and all participants provided informed written consent before participation in this study.

### Learning Materials

#### Selection of Words for the Learning Activity

As the learning contents, 120 well-known Japanese words were selected that are generally written in kana (syllabograms), as most Japanese people cannot read written kanji (morphograms). The words were primary school level that are familiar and easy to image for Japanese people. Actually, the words had high familiarity and imaginability values of >5.7 on average on a seven-point scale (Amano and Kondo, 1999; Sakuma et al., 2005; Table 1). The 120 words were divided into six sets of 20 words each. We confirmed that the lexical properties of the words were matched among the sets based on the results of the Kruskal–Wallis test, where there were no significant differences in the number of characters (*p* = 0.20), number of morae (*p* = 0.98), familiarity values (*p* = 0.50), and imaginability values (*p* = 0.73) across the sets. In the learning activity, each participant learned two sets of words that were randomly selected across participants.

**Table 1:** Lexical properties of word sets.

Lexical property	Set A	Set B	Set C	Set D	Set E	Set F	Kruskal–Wallis test
Number of characters	2.2 ± 0.5	2.1 ± 0.4	2.2 ± 0.6	2.0 ± 0.5	2.3 ± 0.5	2.0 ± 0.5	*p* = 0.20
Numbers of morae	3.5 ± 0.5	3.5 ± 0.5	3.5 ± 0.5	3.5 ± 0.5	3.4 ± 0.5	3.4 ± 0.6	*p* = 0.98
Familiarity values	5.8 ± 0.5	5.7 ± 0.9	6.0 ± 0.5	5.9 ± 0.6	5.8 ± 0.7	6.0 ± 0.5	*p* = 0.50
Imaginability values	5.8 ± 0.6	5.7 ± 0.7	5.9 ± 1.0	5.7 ± 0.7	5.8 ± 0.7	5.7 ± 0.6	*p* = 0.73

#### Stimuli for EEG Experiment

Forty kanji words that each participant wrote in the learning activity (i.e., 20 with an ink pen and 20 with a digital pen) were used as the prime stimuli in the repetition priming paradigm. Each prime stimulus was followed by the target stimuli, which comprised words written in Japanese syllabograms (kana). According to the type of target word, two conditions were set up: repetitive, where the target stimulus represented the reading of the prime stimulus (i.e., semantically and phonologically identical), and non-repetitive, where the target stimulus did not represent the reading of the prime stimulus (i.e., semantically and phonologically different). Trials under the non-repetitive condition used kanji words from one set and readings from the other sets (e.g., pair of kanji words of set A and readings of set B). To prevent unwanted influences from phonological and semantic priming effects on the non-repetition condition, two evaluators checked the presence of phonological similarity and semantic relationships between the prime and target words in each pair. Finally, the word pairs that the both evaluators judged as having no phonological similarity or semantic relationships were adopted. Each kanji word learned by each participant were presented four times as the prime stimulus throughout the experiment: two times for the repetitive condition and two times for the non-repetitive condition. Hence, the participants underwent 160 trials in total: 40 trials with the repetitive condition and 40 trials with the non-repetitive condition in which the prime stimuli were the words learned with a digital pen, as well as 40 trials with the repetitive condition and 40 trials with the non-repetitive condition in which the prime stimuli were the words learned with an ink pen.

### Experimental Procedures

**Figure 2:**
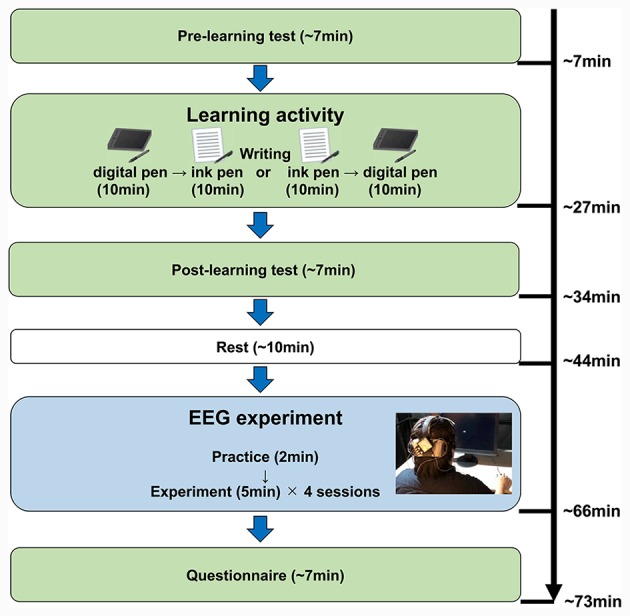
Experimental steps. This chart shows the detailed order of the experimental steps. First, the participants completed a pre-learning test of about 7 min. For the learning activity, the participants learned the kanji notations by writing with each device (10 min each). The order of device use was randomized. Then, the participants completed the post-learning test. After 10-min rest, the electroencephalography (EEG) experiment was conducted for about 22 min. Finally, they answered the questionnaire (about 7 min).

The experiment flow was as follows: (1) pre-learning test; (2) learning activity; (3) post-learning test; (4) EEG measurement; and (5) questionnaire survey (Figure 2). The protocols for each of these steps are described below.

#### Pre- and Post-learning Tests

To investigate the effects of the learning activity on test performance, the participant’s ability to read the selected kanji words was tested both before and after learning. The test sheet given to the participants contained all of the kanji words and the participants answered by writing the correct reading of the words in kana. The words already known by the participant before the learning session in the pre-learning test (Supplementary Figure S1) were excluded from further analyses of both performance and the N400.

#### Learning Activity

For each participant, two sessions were conducted in the learning activity: a digital pen session, during which the participants wrote 20 words of one set repeatedly with a digital pen on a tablet, and an ink pen session, during which they wrote 20 words of the other set with an ink pen on papers. Each session with a given pen-type lasted 10 min. The two sessions were performed in random order among the participants. There was a rest period between the two sessions.

Twenty pairs of kanji words and the corresponding readings (written with kana syllabograms) were written on each learning sheet. The participants were asked to copy the kanji words and to memorize the readings and were told that the readings after the learning activity would be tested.

For the digital pen learning session, a PDF file of the learning sheet was displayed to the participants on a tablet (Cintiq 13HD Creative Pen Display DTK-1301; Wacom Co., Limited, Tokyo, Japan), while the participants wrote with a digital pen (Propen; Wacom). For the ink pen session, the learning sheet was physically placed on a tablet (Intuos Pro Large PTH-851; Wacom) and the participants were instructed to write with an ink pen (Wacom).

#### EEG Measurement

**Figure 3:**
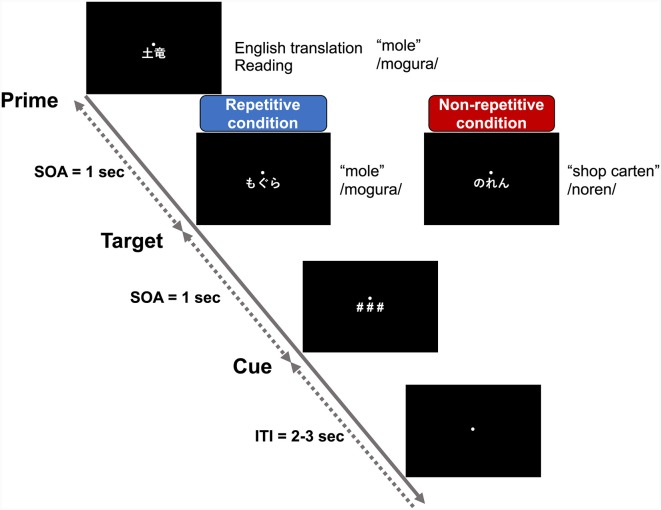
Schematic representation of the repetition priming paradigm. The prime stimuli were words written in morphograms (kanji), which the participants wrote in the learning activity. The target stimuli were words written in syllabograms (kana). In the repetitive condition, the target words were semantically and phonologically identical to the prime stimuli, but distinctly different in the non-repetitive condition. After presentation of the cue (###), the participants answered whether the reading of the target was correct or not by clicking a mouse. The prime, target, and cue were presented with a stimulus-onset asynchrony of 1 s. The intertrial interval between the offset of the cue and the onset of the next prime was randomly set at 2–3 s.

The experiment described in “Stimuli for EEG Experiment” section was conducted while recording the EEG signals. Briefly, the prime, target, and cue (###) were presented continuously with each stimulus-onset asynchrony set to 1,000 ms (Figure 3). The presentation duration for the prime and target stimuli was 300 ms, while that of the cue was 500 ms. The participant was asked to read the prime (kanji) and target (kana) words silently and to answer whether the readings of the prime and target stimuli matched or not by clicking the computer mouse after presentation of the cue. The prime for the next trial was presented 2,000–3,000 ms after the cue onset.

EEG and electrooculography (EOG) signals were measured continuously using an eight-channel wearable EEG device (Polimate mini AP108; Miyuki Giken Co. Limited, Tokyo, Japan). The dry midline electrodes (Unique Medical Co. Limited, Tokyo, Japan) Fz, Cz, and Pz were used according to the International 10-20 system. In addition, to detect the artifacts of eye movements and blinks, and to reject the noise components from the EEG signals, an electrode was placed on the upper and right sides of the left eye to measure the vertical and horizontal EOG components. All signals were sampled at 500 Hz with the use of the left earlobe as the ground and the right earlobe as the reference.

#### Questionnaire Survey

After completing the EEG measurements, the participants responded to questionnaires concerning the use of digital pens on a daily basis as well as the familiarity with the devices used in the learning activity. Each participant was asked to choose either the digital pen or the ink pen with respect to: (1) which they felt required a greater workload; (2) was more enjoyable; (3) easier to memorize; and (4) easier to write with.

### Data Analyses

#### Performance

The difference between the number of correct answers on the reading test before and after the learning activity was recorded as the number of words memorized. To assess the differences between groups and devices, two-factor, mixed-design analysis of variance was performed with factors of familiarity (familiar vs. unfamiliar group) and learning device (digital vs. ink pen).

#### N400

Analysis of the EEG and EOG signals was conducted using MATLAB (MathWorks Inc., Natick, MA, USA) and the EEGLAB toolbox^1^. A bandpass filter of 0.2–30 Hz was applied to the measured EEG and EOG signals, and artifact components, mainly caused by eye movements and blinks, were excluded from the EEG signals using noise reduction processing with artifact subspace reconstruction and independent component analysis. Next, the signals from 100 ms before to 800 ms after target onset were averaged for each condition (repetitive vs. non-repetitive) and each channel, and corrected using 100 ms before target onset as a baseline. Trials exceeding ±80 μV on the Fz, Cz, and Pz channels, those exceeding ±100 μV on the vertical and horizontal EOG channels, and those of words correctly adjusted based on preliminary reading tests were excluded from the average.

The Cz or Pz electrode with a large repetition priming effect on N400 was used as an analytical target for each participant and each learning device. The recorded N400 waves had an average amplitude of 300–450 ms after target onset.

Effects of familiarity and learning device on the repetition priming effect of the N400 were analyzed using the same two-factor analysis of variance described in “Performance” section. Furthermore, as a *post hoc* test, the *t*-test was conducted within each group to identify differences in the repetition priming effect between the two devices.

#### Questionnaire Survey

The *χ*^2^ test was performed to determine whether the familiar group differed from the unfamiliar group regarding the proportion of participants who responded that workload, enjoyability, ease of memorization, and ease of writing were greater with the use of a digital pen vs. an ink pen.

## Results

### Learning Activity and Pre/Post-learning Tests

**Figure 4:**
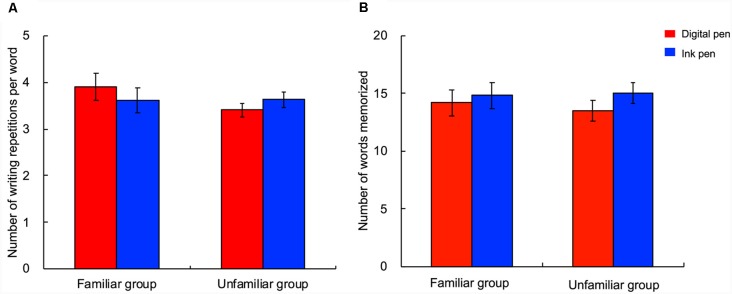
Number of writing repetitions per word and number of words memorized. The number of writing repetitions per word during the learning activity **(A)** and the number of memorized words **(B)** were not affected by either the writing device or familiarity with the digital device. Each bar shows the grand average of the participants. The error bar represents the standard error. The red and blue bars indicate the use of a digital and ink pen, respectively.

Regarding the number of writing repetitions per word, there was no main effect of the participant group (*F*_(1,26)_ = 0.78; *p* = 0.39, partial *η*^2^ = 0.03), main effect of the learning device (*F*_(1,26)_ = 0.04; *p* = 0.84, partial *η*^2^ = 0.00), or interaction between the two factors (*F*_(1,26)_ = 2.98; *p* = 0.10, partial *η*^2^ = 0.10; Figure 4A). Similarly, with regard to the number of words memorized, there was no main effect of the participant group (*F*_(1,26)_ = 0.03; *p* = 0.87, partial *η*^2^ = 0.00), main effect of the learning device (*F*_(1,26)_ = 2.94; *p* = 0.10, partial *η*^2^ = 0.10), or interaction (*F*_(1,26)_ = 0.50; *p* = 0.49, partial *η*^2^ = 0.02; Figure 4B). Thus, performance in learning was not affected by either familiarity or the learning device used.

### EEG Experiment

**Figure 5:**
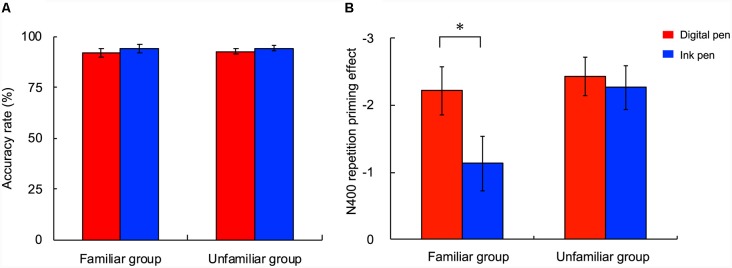
Accuracy rate and repetition priming effect of the N400 in the EEG experiment. **(A)** The accuracy rate was not affected by the learning device or group. **(B)** The repetition effect of the N400 was significantly greater for words learned with the digital pen (red) than with the ink pen (blue) in the familiar group, but not in the unfamiliar group. The bars indicate mean amplitudes of the difference-event-related potential (ERP) from 300 to 450 ms (i.e., non-repetitive condition minus repetitive condition) between groups. The error bars represent the standard error. *p < 0.05.

Regarding the accuracy rates for the task during the EEG experiment, there was no main effect of the participant group (*F*_(1,26)_ = 0.10; *p* = 0.76, partial *η*^2^ = 0.00) or learning device (*F*_(1,26)_ = 0.02; *p* = 0.90, partial *η*^2^ = 0.00) and no interaction between the participant group and learning device (*F*_(1,26)_ = 0.02; *p* = 0.90, partial *η*^2^ = 0.00; Figure 5A).

**Figure 6:**
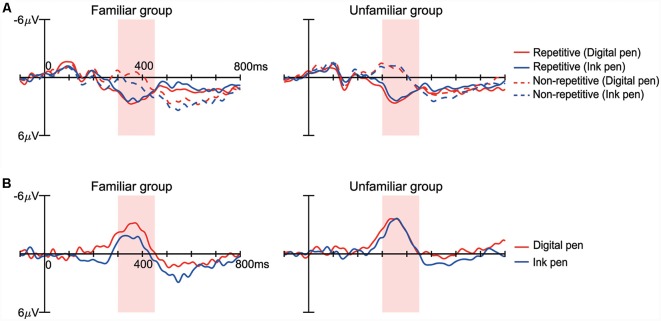
ERPs measured in the repetition priming paradigm. **(A)** The ERPs from approximately 300–450 ms varied between the repetitive condition (solid line) and non-repetitive condition (dashed line) in both the familiar and unfamiliar groups. The red and blue lines indicate the digital and ink pen condition, respectively. **(B)** The difference in waveforms obtained by subtracting the repetitive condition from the non-repetitive condition had a negative peak at approximately 300–450 ms. In the familiar group, the amplitude was smaller for the ink pen (blue) than the digital pen (red), while there was no difference in the unfamiliar group.

For each participant group and device, the N400 (ERP; 350–450 ms) with the repetitive condition was smaller than with the non-repetitive condition (Figure 6A). With specific regard to the repetition priming effect of the N400 (i.e., difference between N400 amplitudes measured under non-repetitive and repetitive conditions; Figure 6B), a main effect of the learning device was observed (*F*_(1,26)_ = 4.78; *p* = 0.04, partial *η*^2^ = 0.16). That is, the repetition priming effect was greater with the use of a digital pen vs. an ink pen. In contrast, there was no main effect of the participant group (*F*_(1,26)_ = 2.81; *p* = 0.11, partial *η*^2^ = 0.10) or interaction between the participant group and learning device (*F*_(1,26)_ = 2.58; *p* = 0.12, partial *η*^2^ = 0.09).

When the differences between the learning devices were examined within each participant group as a *post hoc* test (Figure 5B), the repetition priming effect was significantly greater in the familiar group with the use of a digital pen vs. an ink pen (*t*_(10)_ = −2.37; *p* = 0.02, *d* = 0.90), while the effect was similar between pen types in the unfamiliar group (*t*_(16)_ = −0.47; *p* = 0.32, *d* = 0.13).

To determine whether the difference in the N400 effect between the familiar and unfamiliar groups resulted from differences in the ratio of men to women (i.e., familiar group: 10 men and one woman; unfamiliar group: 10 men and seven women), the data of only men were also analyzed because the number of men was the same in the two groups. The results showed that the same effect was obtained from all participants; that is, the familiar group had a greater priming effect of the N400 for the words learned with a digital pen than those with an ink pen, while in the unfamiliar group, there was no effect by the learning device (Supplementary Figure S2). Therefore, the difference obtained among all participants likely did not result from differences in the sex ratio.

### Questionnaire Survey

**Figure 7:**
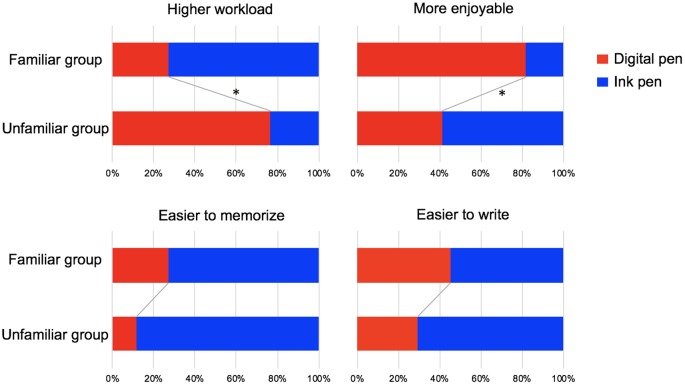
Results of the questionnaire survey. Regarding the number of participants who answered “digital pen” (red) and “ink pen” (blue) to each survey item, the proportions differed significantly between the familiar and unfamiliar groups with respect to the degree of workload (*p* = 0.01) and enjoyability (*p* = 0.03). A greater number of participants in the familiar group (8 of 11) answered that “the use of the ink pen required a greater workload,” whereas most participants in the unfamiliar group (13 of 17) answered that “the use of the digital pen required a greater workload.” In addition, most participants in the familiar group (9 of 11) answered that “the digital pen was more enjoyable to use,” whereas a slight majority of participants in the unfamiliar group (10 of 17) answered that “the ink pen was more enjoyable to use.” There was no significant difference between groups regarding memorability and ease of writing. *p < 0.05.

Figure 7 displays the tallied results of the participants’ answers to the survey questions concerning workload, enjoyability, ease of memorization, and ease of writing. Regarding the number of participants who answered “digital pen” and “ink pen” to each survey item, the proportions differed significantly between the familiar and unfamiliar groups with respect to the degree of workload (*χ*^2^ = 6.60;* p* = 0.01, *φ* = 0.49) and enjoyability (*χ*^2^ = 4.50; *p* = 0.03, *φ* = 0.40). In the familiar group, a larger number of participants (8 of 11) than in the unfamiliar group answered that “the use of the ink pen required a greater workload.” In the unfamiliar group, most participants (13 of 17) answered that “the use of the digital pen required a greater workload.” In addition, most participants in the familiar group (9 of 11) responded that “the digital pen was more enjoyable to use.” Conversely, in the unfamiliar group, a slight majority of participants (10 of 17) responded that “the ink pen was more enjoyable to use.” Meanwhile, no significant differences were observed between the two participant groups with respect to memorability and ease of writing.

## Discussion

In the present study, the modulation of electrophysiological signals was investigated by writing words with a digital pen vs. a conventional ink pen, while comparing between participants familiar vs. unfamiliar with the digital device. Regarding the performance level, there were no differences in the number of writing repetitions for each word over a 10-min period and the number of words memorized afterward between the learning devices in both groups, or in the accuracy rate of the EEG experiment. In contrast, the repetition priming effect of the N400 detected immediately after learning showed the difference between the learning devices. In the familiar group, the repetition priming effect of the N400 was significantly greater for the words that were learned by writing with a digital pen vs. an ink pen, but not in the unfamiliar group. The dissociation of performance and the N400 effect might be due to differences in the sensitivity to detect learning effects. That is, the N400 effect was more sensitive to early learning compared with the behavioral assessment (McLaughlin et al., 2004). Therefore, our result indicates that when the participants are familiar with the use of digital pens, learning with the ink pen gave rise to lesser effect on brain activity.

From these results, the question arises why the use of a digital device might affect brain activity. Our questionnaire survey indicated that more participants in the familiar group found it fun to use the digital pen and felt less workload when using the device, as compared with the participants in the unfamiliar group. Previous studies have reported that mood can affect learning (Nadler et al., 2010; Bakic et al., 2014) and language processing (Federmeier et al., 2001; Vissers et al., 2010, 2013; Chwilla et al., 2011). Therefore, the difference in the N400 effect between the learning devices might be caused by differences in mood while using a particular device (i.e., less enjoyable and higher workload when writing with an ink pen). However, the data obtained in this study were insufficient for further investigation, which is a limitation to this study. Nonetheless, further research on this topic is warranted.

## Data Availability

All datasets generated for this study are included in the manuscript and/or the Supplementary Files.

## Ethics Statement

The study was conducted after approval was obtained from the Bioinformatics Ethics Committee of the National Institute of Information and Communications Technology, and all participants provided informed written consent in advance regarding their participation in this study.

## Author Contributions

AI, YN, KN, YY, and AK designed the experiment. KN prepared the materials and experimental equipment. KN, KO, and KI conducted the experiments. KN and KO analyzed the data. KO and AI wrote the first draft of the manuscript, and all authors revised the draft and approved the final version.

## Conflict of Interest Statement

AK and KI are employees of Wacom Co., Ltd. The remaining authors declare that the research was conducted in the absence of any commercial or financial relationships that could be construed as a potential conflict of interest.

## Footnotes

^1^https://sccn.ucsd.edu/eeglab/index.php
