# Supplementary material for: Differences in Brain Activity After Learning With the Use of a Digital Pen vs. an Ink Pen—An Electroencephalography Study
Source: Front Hum Neurosci. 2019 Aug 9;13:275. doi: 10.3389/fnhum.2019.00275 (PMC6697244; doi:10.3389/fnhum.2019.00275)
Supplement: Supplementary file 1 [file Data_Sheet_1.docx]

Supplementary Materials


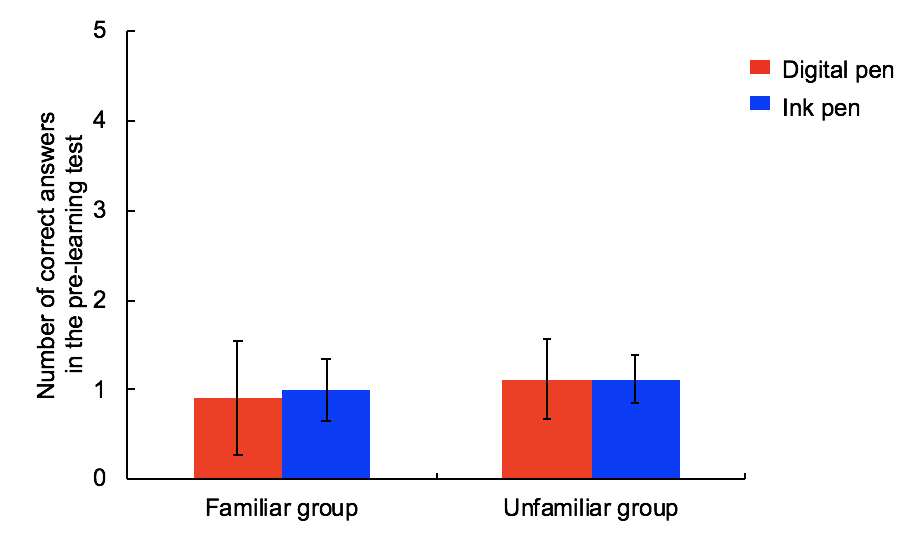


**Supplementary Figure 1.** The numbers of correct answers in the pre-learning test obtained by comparing between words used for the learning session with a digital pen and those used for the learning session with an ink pen and between participant groups. There were no main effects of the learning device (*F* (1, 26) = 0.02; *p* = 0.90, partial *η^2^* = 0.00) and participant groups (*F* (1, 26) = 0.10; *p* = 0.76, partial *η^2^* = 0.00) and no interaction between the learning device and participant group (*F* (1, 26) = 0. 02; *p* = 0.90, partial *η^2^* = 0.00). The mean number of correct answers among participants is depicted. The error bars indicate the standard error.


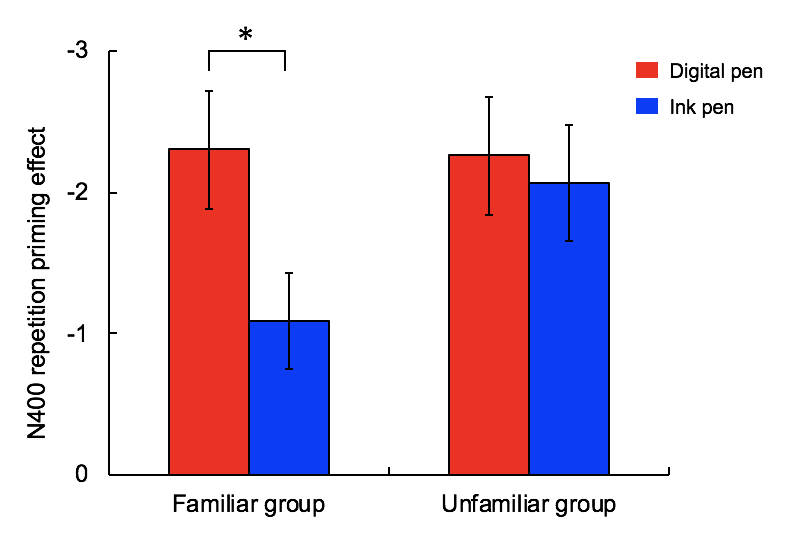


**Supplementary Figure 2.** The N400 repetition priming effect of only men (familiar group: N=10; unfamiliar group: N=10). As the main effect of the learning device (*F* (1, 18) = 4.84; *p* = 0.04, partial *η^2^* = 0.21), the repetition priming effect was greater for words that were written with a digital pen than for those with an ink pen. In contrast, there was no main effect of the participant group (*F* (1, 18) = 1.01; *p* = 0.33, partial *η^2^* = 0.05) and no interaction between the learning device and participant group *(F* (1, 18) = 2.50; *p* =0.13, partial *η^2^* = 0.12). When the differences between the learning devices were examined within each participant group as a post-hoc test, the familiar group had a significantly greater repetition priming effect for words written with a digital pen than an ink pen (*t* (9) = −2.50; *p* = 0.02, *d* = 0.92), while in the unfamilar group, there was no diifference between the digital and ink pens (*t* (9) = −0.47; *p* = 0.32, *d*=0.16). The mean number of correct numbers among participants is depicted. The error bars indicate the standard error. **p* < 0.05.
